# Characterization of the Biophysical Properties and Cell Adhesion Interactions of Marine Invertebrate Collagen from *Rhizostoma pulmo*

**DOI:** 10.3390/md21020059

**Published:** 2023-01-19

**Authors:** Ian P. Smith, Marco Domingos, Stephen M. Richardson, Jordi Bella

**Affiliations:** 1 Division of Cell Matrix Biology and Regenerative Medicine, School of Biological Sciences, Faculty of Biology, Medicine and Health, Manchester Academic Health Science Centre, University of Manchester, Manchester M13 9PT, UK; 2 Department of Mechanical, Aerospace and Civil Engineering, Faculty of Science and Engineering and Henry Royce Institute, University of Manchester, Manchester M13 9PY, UK

**Keywords:** marine collagen, jellyfish collagen, *Rhizostoma pulmo*, collagen fibrillogenesis, cell adhesion, heparan sulfate

## Abstract

Collagen is the most ubiquitous biomacromolecule found in the animal kingdom and is commonly used as a biomaterial in regenerative medicine therapies and biomedical research. The collagens used in these applications are typically derived from mammalian sources which poses sociological issues due to widespread religious constraints, rising ethical concern over animal rights and the continuous risk of zoonotic disease transmission. These issues have led to increasing research into alternative collagen sources, of which marine collagens, in particular from jellyfish, have emerged as a promising resource. This study provides a characterization of the biophysical properties and cell adhesion interactions of collagen derived from the jellyfish *Rhizostoma pulmo* (JCol). Circular dichroism spectroscopy and atomic force microscopy were used to observe the triple-helical conformation and fibrillar morphology of JCol. Heparin-affinity chromatography was also used to demonstrate the ability of JCol to bind to immobilized heparin. Cell adhesion assays using integrin blocking antibodies and HT-1080 human fibrosarcoma cells revealed that adhesion to JCol is primarily performed via β1 integrins, with the exception of α2β1 integrin. It was also shown that heparan sulfate binding plays a much greater role in fibroblast and mesenchymal stromal cell adhesion to JCol than for type I mammalian collagen (rat tail collagen). Overall, this study highlights the similarities and differences between collagens from mammalian and jellyfish origins, which should be considered when utilizing alternative collagen sources for biomedical research.

## 1. Introduction

Collagen is the most abundant protein found in all animals, from mammals to invertebrates. It constitutes the most important molecular component of the extracellular matrix (ECM) in most tissues. To date, at least 28 collagen types, coded by more than 40 genes, have been confirmed in vertebrates [1,2]. These different collagens provide structural and mechanical stability to animal tissues and facilitate a range of biological functions, such as cell migration, proliferation and differentiation. Thus, collagens are highly attractive substrates for use in biomedical research and medical device coating [1,3,4].

Collagens can be fibrillar or non-fibrillar depending on their supramolecular structure [5,6]. The major fibrillar collagens (types I–III) are the most abundant in mammals and are the primary types used in biomedical, cosmetic and nutraceutical applications [7,8,9,10,11,12]. These collagens are predominantly sourced from discarded porcine and bovine hides, tendons, bones and hooves from commercial abattoirs [13]. However, religious constraints and increasing social concern over the ethics of cultivating animal products have led to a rise in research into alternative collagen sources [14].

In addition to the social implications of these mammalian sources, there are significant health concerns to be acknowledged after the outbreak of bovine spongiform encephalopathy in the UK, as well as the persistent risk of other transmissible spongiform encephalopathies or zoonotic diseases [15]. The reported immunogenic responses to bovine material provide a further incentive to use non-mammalian collagens for biomedical applications [16].

Several alternative collagens have been discovered or developed in the form of recombinant collagens produced by genetically modified E. coli strains, yeast collagens, mammalian cells and even plant cells. However, the application of collagen from these sources is limited by low yields, high production costs and the difficulties in posttranslational modification [4,8,9].

Marine invertebrates present an attractive novel biomaterial source, as reflected in the steady rise in publications over the last 20 years [17]. In addition to the large array of bioactive peptides and other marine biomolecules that have been reported, marine invertebrate collagens are increasingly being recognized as alternatives to mammalian collagens [18,19,20,21,22,23].

Jellyfish, in particular, stand out as a particularly attractive marine invertebrate collagen source due to their high proportion of collagen relative to insoluble extracts, their lack of calcified tissues and the similarity in morphology and function to mammalian fibrillar collagens [24]. In addition to their biological suitability, there are potential benefits to harvesting jellyfish given the significant socioeconomic impact of coastal blooms on beach tourism, marine ecosystems and fishery stocks [25,26,27].

Several studies claim that jellyfish-derived collagens from various species are analogous to specific collagen types found in mammals, and whilst this may not be entirely true due to differences in amino acid composition, their use as scaffolds in vivo has shown some success in the regeneration of cartilage, bone and vascular tissue [28,29,30]. Jellyfish collagen has also been used in 2D and 3D in vitro models of mesenchymal stem cells (MSCs), induced pluripotent stem cells (iPSCs), chondrocytes, chondroprogenitors, osteoblasts, fibroblasts and ovarian cancer cell lines [31,32,33,34,35,36,37,38].

Recent reports on collagen derived from the Mediterranean basin scyphozoan *Rhizostoma pulmo* include only a partial characterization of the collagen itself and have focused primarily on the construction of 3D scaffolds. Khalturin et al. have shown, through genome sequencing of two cnidarian species, that jellyfish have a low proportion of genetic homogeneity between species—approximately the same as between humans and sea urchins [39]. Therefore, the peptide sequences of collagens from different jellyfish are unlikely to be identical and separate characterizations of the collagens derived from each species are necessary before their potential applications can be fully appreciated.

The purpose of this study was to provide a basic characterization of the physicochemical properties of collagen derived from *R. pulmo* (JCol), and to explore some of its cell adhesion interactions. In addition to the fundamental characterization of the molecular composition, secondary structure and melting temperature, we show here that JCol is fibrillar and explore the relationship between the extent of fibril formation (fibrillogenesis) and ionic strength over time. We have also investigated the cell adhesion interactions of human fibroblasts, fibrosarcoma cells and MSCs with JCol. Using chromatography and cell assays, we confirm that there is an adhesion interaction between heparin and JCol which is not observed with type I mammalian collagen. The differences in integrin binding to JCol and mammalian collagen have also been investigated using inhibitory antibodies, revealing an absence of α2β1 integrin binding motifs on JCol.

## 2. Results

### 2.1. Molecular Characterization of Jellyfish Collagen

#### 2.1.1. SDS-PAGE

**Figure 1:**
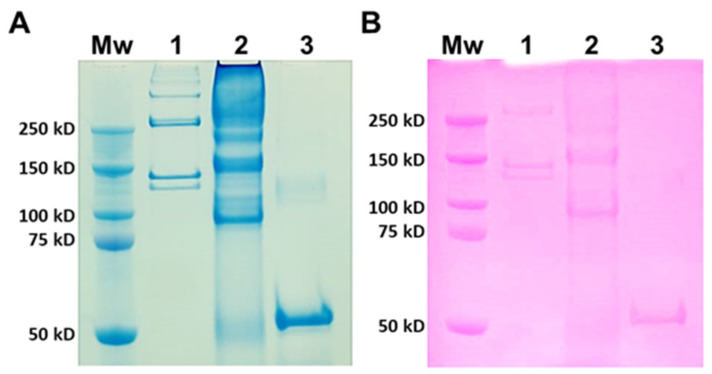
SDS-PAGE analysis of rat tail collagen (RTC) and JCol. Protein bands are stained with Coomassie Blue (**A**) and Periodic Acid-Schiff (**B**). Lanes (panels (**A**,**B**)) are molecular weight marker (Mw), RTC (1), JCol (2) and horseradish peroxidase positive control (3).

Type I collagen from rat tails was used as a mammalian comparison to JCol for SDS-PAGE (Figure 1). Type I collagen is a heterotrimer composed of two α1 chains and one α2 chain, with typical molecular weights of 129 kD and 139 kD, respectively [40]. The larger molecular weight bands signify the presence of β dimers (approximately 260 kD) or γ trimers (>350 kD) of α chains [41]. JCol shows a polydisperse sample with potential α chains present at 150 kD and 160 kD. These apparent molecular weights are slightly higher than those observed in the SDS-PAGE analysis of mammalian collagens. An additional smaller protein band is visible at 90 kD, which is not comparable to any band from mammalian type I collagen and may either be a collagen specific to *R. pulmo* or found in other marine invertebrates. This smaller protein band may also be an accessory collagen to the main fibrillar collagen chains. The faint bands observed immediately above this major band, at 90 kD, may be collagen fragments incurred during the purification process. The Periodic Acid-Schiff (PAS) staining of an identical gel shows a degree of glycosylation present in all of the proteins from both samples that is relatively proportional to the amount of protein loaded.

#### 2.1.2. Circular Dichroism Spectroscopy

**Figure 2:**
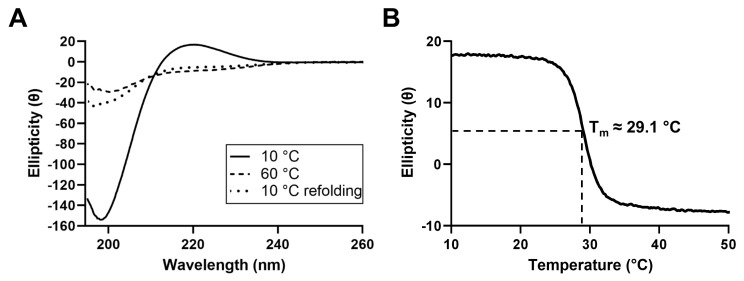
CD spectra of JCol at 10 °C, 60 °C and 10 °C immediately after melting (**A**). Change in ellipticity of JCol at a fixed wavelength of 220 nm under increasing temperature from 10 °C to 50 °C at a rate of 0.5 °C/min (**B**).

In order to characterize the secondary structure of the proteins present in JCol, circular dichroism (CD) spectroscopy was performed (Figure 2). The CD spectrum of JCol shows a peak in ellipticity at 220 nm and a trough at 198 nm, which are typical for the triple-helical conformation of collagen [42,43].

The decrease in ellipticity at 220 nm during heating indicates the loss of the triple-helical structure and the separation of the α chains. The melting temperature, defined as the temperature at which 50% of the collagen in the sample is denatured, was calculated to be approximately 29.1 °C. This melting temperature is considerably lower than those of mammalian collagens (approximately 37 °C), which is to be expected given the more temperate habitat of *R. pulmo* and, therefore, the lower physiological temperature required for tissue remodeling in vivo [44]. Partial refolding of the triple-helical structure was observed when the CD spectra were measured immediately after cooling to 10 °C, indicating the ability of this collagen to gradually regain its natural conformation after heat denaturation.

#### 2.1.3. Heparin-Affinity Chromatography

**Figure 3:**
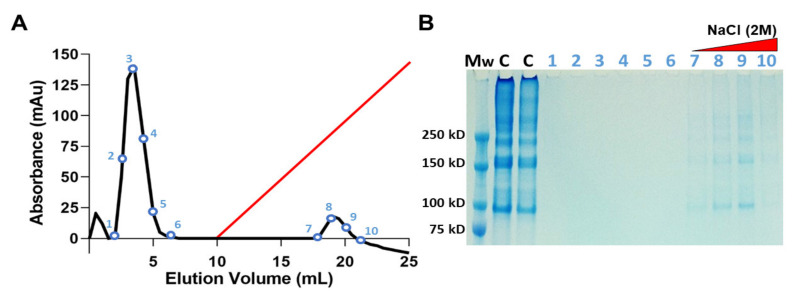
Heparin-affinity chromatography of JCol in 10 mM sodium phosphate at pH 7.4. Elution profile shows a peak of heparin-bound protein detected during linear elution gradient (red) using 2 M NaCl at a flow rate of 0.5 mL/min (**A**). SDS-PAGE of the NaCl eluted fractions identifies the heparin-bound portion as JCol (**B**). Lanes (left-right) are molecular weight marker (Mw), JCol (C) and eluted fractions (1–10).

The ability of JCol to bind with heparan sulfate (HS) and HS proteoglycans was investigated using heparin-affinity chromatography (Figure 3). A population of heparin-binding proteins retained by the heparin column are shown to elute at approximately 1 M NaCl concentration. These proteins were later verified as JCol using SDS-PAGE. The collagen bands in the eluted fractions appear faint in comparison to the JCol stock solution control, most likely due to the diluting effect of the elution procedure. A large peak eluted from the column before the NaCl gradient was initiated, suggesting that a portion of the original sample did not bind to the immobilized heparin and passed through the column as flow-through. SDS-PAGE analysis of the non-binding fractions did not show any protein bands, possibly due to the low molecular weight of their components.

### 2.2. Jellyfish Collagen Fibrillogenesis

#### 2.2.1. Fibrillogenesis Assay

**Figure 4:**
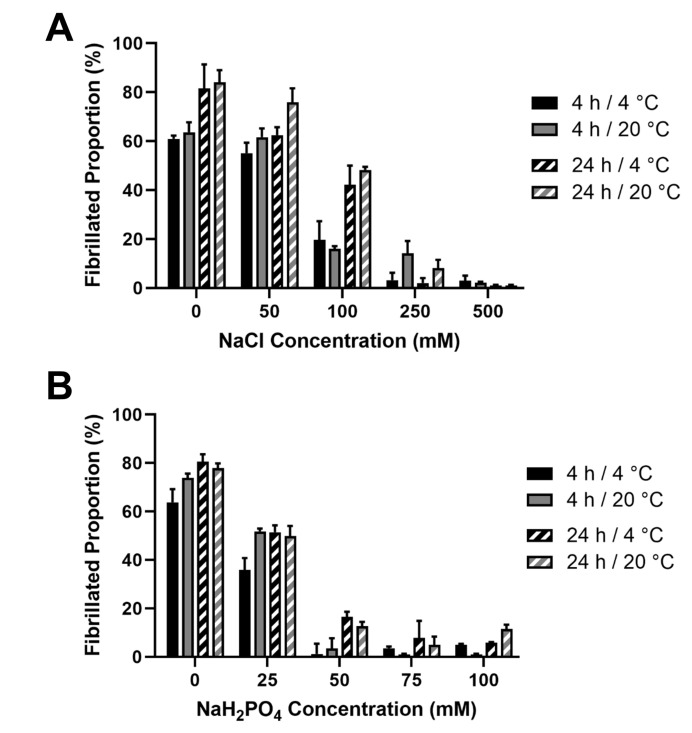
Effect of salt concentration on the proportion of fibril formation by JCol after 4 h and 24 h at 4 °C and 20 °C. Fibrillogenesis under increasing concentrations of NaCl and NaH_2_PO_4_ are shown in panels (**A**,**B**), respectively.

In order to verify the fibrillar nature of the JCol, a fibrillogenesis assay was established. Acid-solubilized JCol was induced to begin fibril formation by neutralizing with 25 mM Tris buffer and 200 mM NaOH in order to achieve a pH of 7.2–7.4. The effects of the ionic strength (by means of NaCl and NaH_2_PO_4_ addition) and temperature (4 °C and 20 °C) were investigated using a BCA assay [45]. It was observed that, for both salts, the proportion of insoluble fibers in a neutral buffered solution decreased drastically with the increasing ionic strength (Figure 4).

This inhibitory effect was notably stronger for NaH_2_PO_4_, which induced a maximal fibrillogenesis inhibitory effect at approximately 50 mM final concentration, in comparison to the 500 mM observed with NaCl. A slight increase in the fibrillated proportion was observed at all ionic strengths when the incubation time was increased from 4 h to 24 h. Altering the incubation temperature (whilst keeping it below the melting point) did not produce any notable effect on the proportion or time taken for the fibers to precipitate. It was observed that the highest proportion of fibrillated protein was approximately 80%, suggesting that potentially 20% of the JCol stock is comprised of non-fibrillar collagens, short collagen fragments resulting from partial digestion or non-collagenous proteins. It is also possible that any non-collagenous proteins present in JCol may have co-precipitated with the fibrillar collagens.

#### 2.2.2. Atomic Force Microscopy

**Figure 5:**
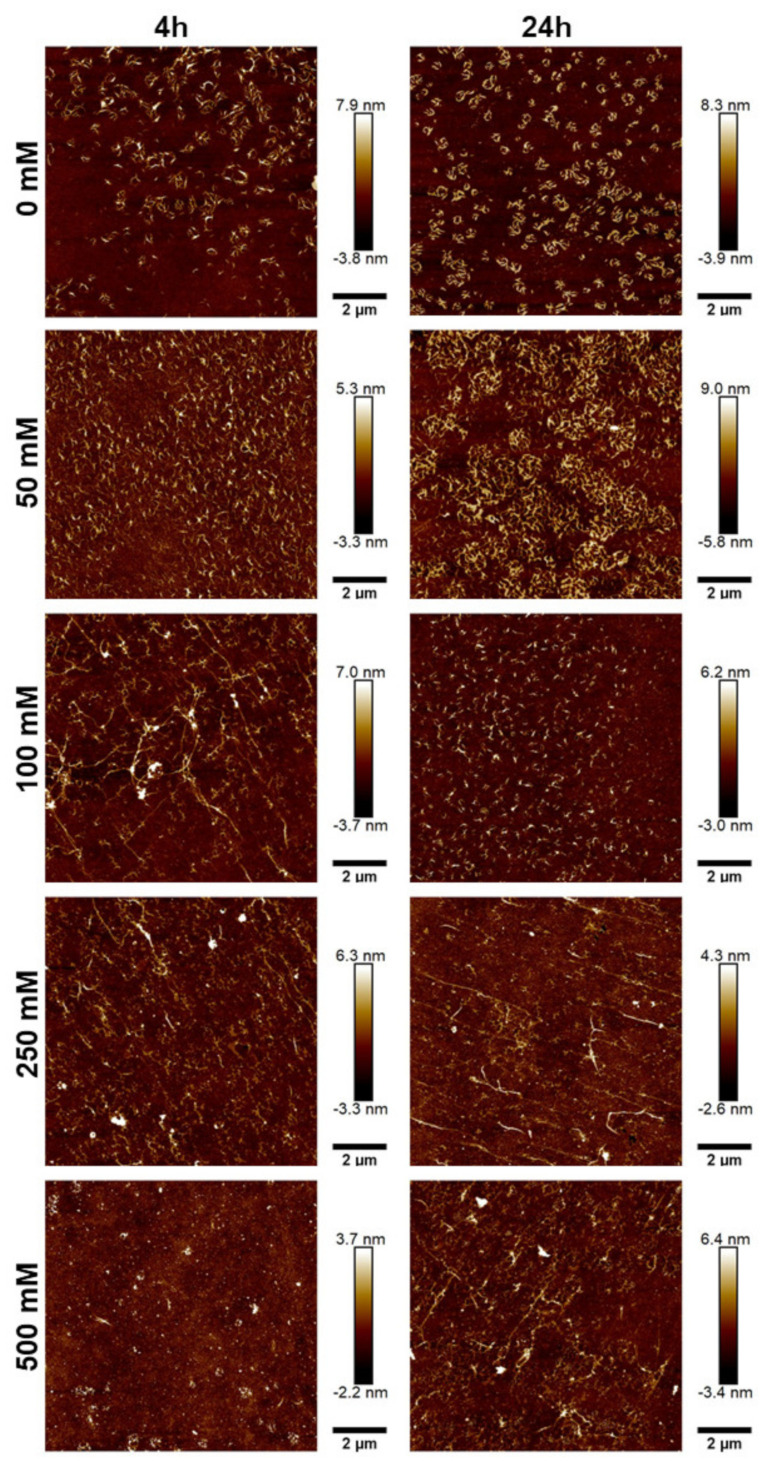
AFM height plots of JCol buffered with increasing NaCl concentrations for 4 h (L) and 24 h (R).

**Figure 6:**
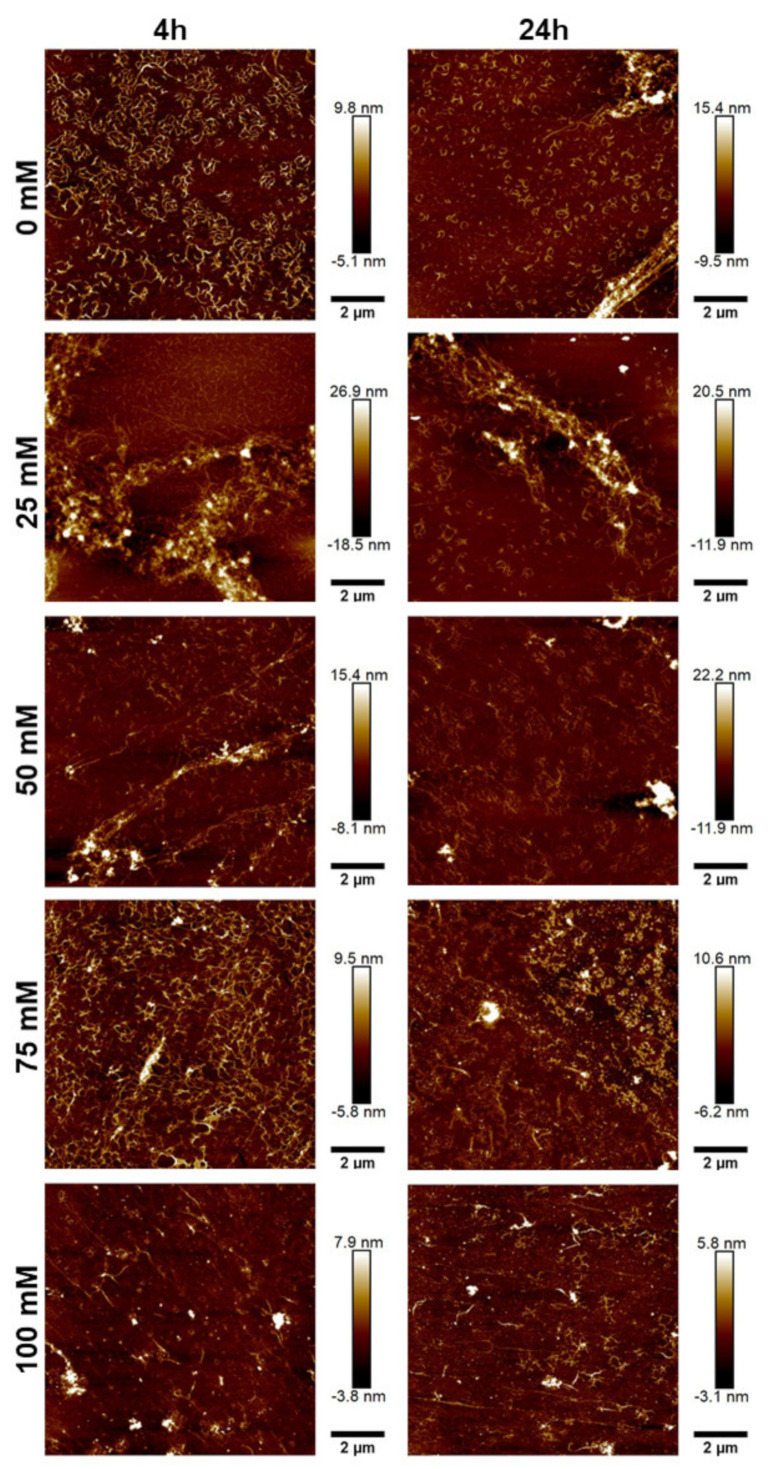
AFM height plots of JCol buffered with increasing NaH_2_PO_4_ concentrations for 4 h (L) and 24 h (R).

To further characterize the morphology of the JCol, atomic force microscopy (AFM) was performed on the salt-buffered collagen solutions used in the fibrillogenesis assay. The effect of increasing the NaCl and NaH_2_PO_4_ concentrations is shown in Figure 5 and Figure 6, respectively.

For both salts, it was observed that an intermediate ionic strength (100 mM NaCl and 25 mM NaH_2_PO_4_) enabled the optimal formation of fibers despite the lower proportion of insoluble collagen available, as measured using the fibrillogenesis assay. At lower salt concentrations, the morphology of the collagen was more ‘aggregated’, showing clusters of branching microfibrils as opposed to the typical long fibers associated with polymeric fibrillar collagen, as found in tissues *in vivo*. Excessive salt concentrations resulted in the loss of long collagen fibers or fibrillar clusters and showed more of a globular or denatured protein appearance, presumably due to strong ionic interactions from the buffer.

NaH_2_PO_4_ was shown to produce larger fibers than NaCl (as measured through height scales), possibly due to the ability of collagen-bound phosphate salts to significantly influence electrostatic interactions and aid in fibril formation [46]. The efficiency of fibrillogenesis was visually determined to be highly variable between samples at different ionic strengths, which negated the usefulness of quantitatively comparing the fiber size between conditions. The prolonged incubation time from 4 h to 24 h only improved fibrillogenesis under suboptimal conditions (high or low ionic strength), and generally did not benefit the fibers that had already formed after 4 h.

### 2.3. In Vitro Culture of Human Fibroblasts and MSCs on Jellyfish Collagen

#### 2.3.1. Proliferation and Spreading Assay

**Figure 7:**
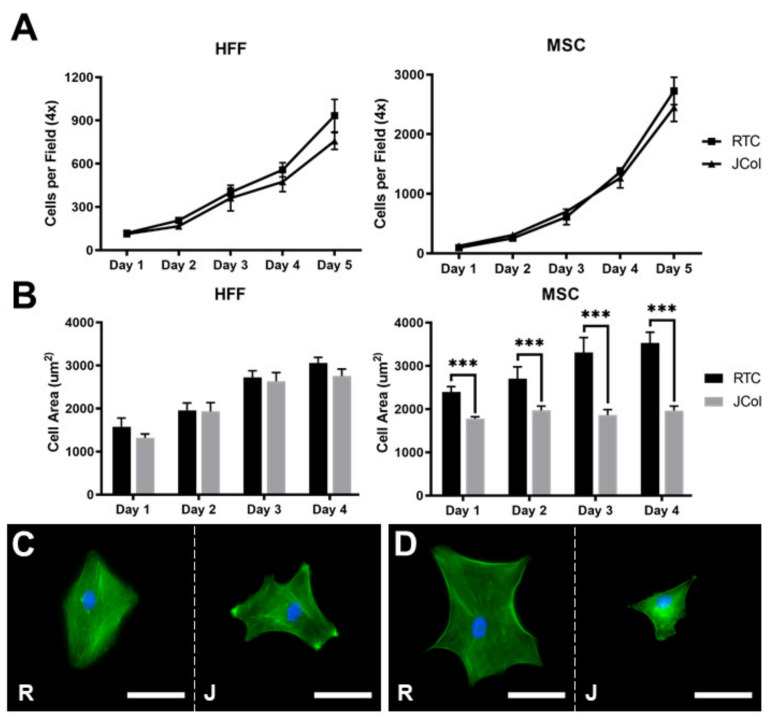
Proliferation curves (**A**) and cell spreading (**B**) of HFFs and MSCs on JCol and RTC. Representative images of HFF (**C**) and MSC (**D**) spreading on RTC (R) and JCol (J) at day 4. Cytoskeleton is stained green using Alexa Fluor™ 488 phalloidin (F-actin) and nuclei are stained blue using Hoescht solution (DNA). Scale bars are 50 µm. Statistical significance was determined by an unpaired two-tailed Student’s *t*-test (*** = *p* < 0.001).

The proliferation and spreading of human foreskin fibroblasts (HFFs) and immortalised human MSCs on 2D JCol and RTC coatings were quantified using fluorescence microscopy (Figure 7). There was no significant difference between the doubling times measured over 5 days for HFFs or MSCs (approximately 33 and 21 h, respectively) on either collagen coating. The extent of cell spreading was also comparable for HFFs on both coatings; however, the MSCs showed a significant increase in cell area at all time-points when cultured on RTC in comparison to JCol. This may suggest that the adhesion motifs utilized by MSCs are present in a lower proportion on JCol than on RTC.

#### 2.3.2. Adhesion Assay

**Figure 8:**
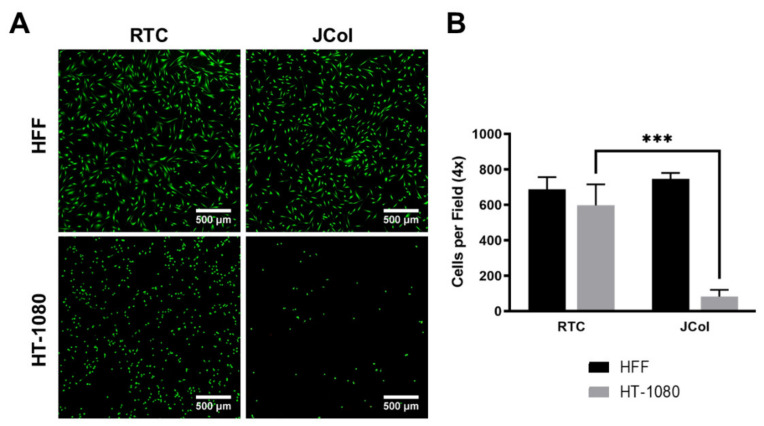
Representative images of HFF and HT-1080 cell adhesion on RTC and JCol after 3 h (**A**). Cells are stained green using Alexa Fluor™ 488 phalloidin (F-actin). Scale bars are 500 µm. Quantification of cells per 4x field of view adhered to both coatings (**B**). Statistical significance was determined by an unpaired two-tailed Student’s *t*-test (*** = *p* < 0.001).

Human fibrosarcoma (HT-1080) cells were used to investigate whether JCol contains sequences which bind to α2β1 integrin, as these cells are known to bind to collagen almost exclusively via this receptor [47,48]. HT-1080 cells also bind to RGD-containing ECM proteins, such as fibronectin, via α5β1 integrin; however, this receptor should not bind to collagen sequences. A drastic reduction in the number of adhered cells (approximately 90%) was observed when culturing HT-1080 cells on JCol in comparison to RTC (Figure 8). This would suggest that JCol does not contain, or has significantly fewer, α2β1 binding motifs than mammalian type I collagens. This reduction was not seen with HFFs, suggesting that other binding mechanisms are responsible for cellular adhesion to JCol.

#### 2.3.3. Integrin and Heparan Sulfate Chain Adhesion Interactions of Jellyfish Collagen

**Figure 9:**
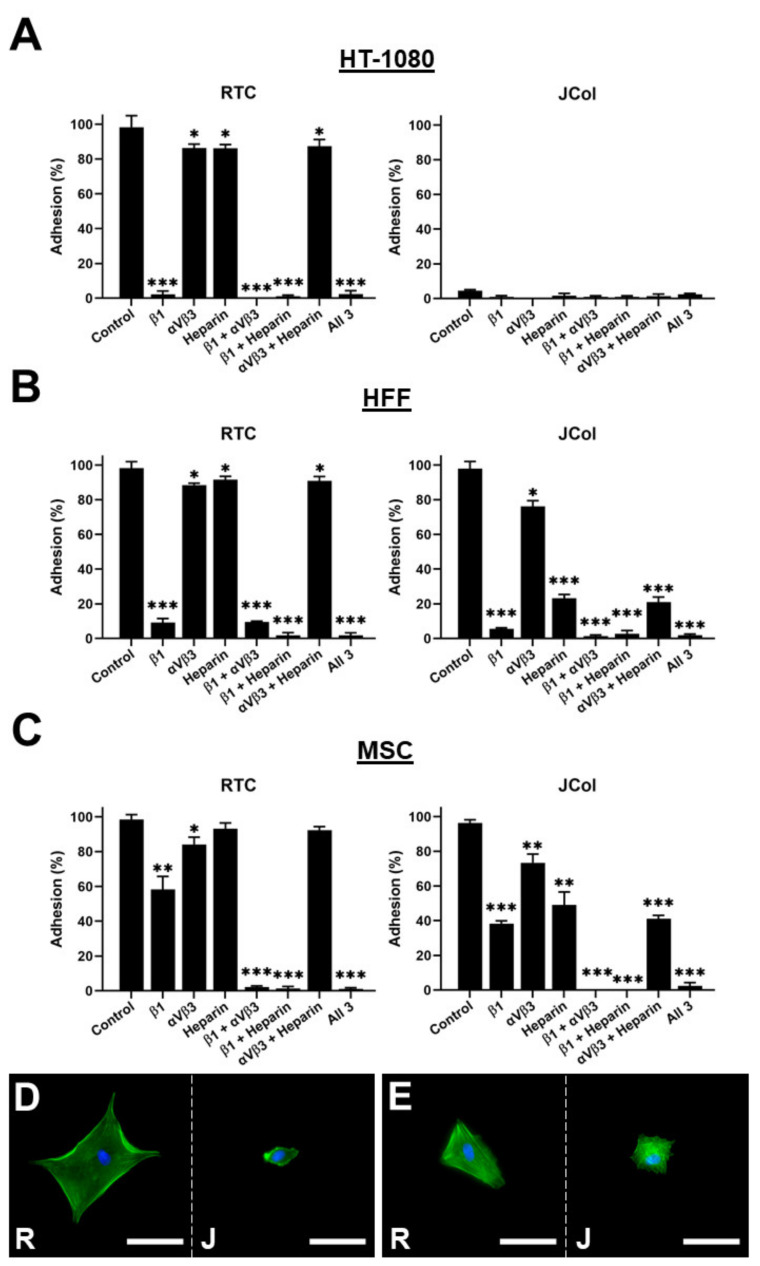
Cell adhesion plots for HT-1080, HFF and MSC cell lines on RTC or JCol with or without binding inhibition to β1 integrin subunit, αVβ3 integrin and heparan sulfate chains (**A**–**C**). Representative images of HFFs (**D**) and MSCs (**E**) on RTC (R) and JCol (J) treated with heparin sodium salt. Cytoskeleton is stained green using Alexa Fluor™ 488 phalloidin (F-actin) and nuclei are stained blue using Hoescht solution (DNA). Scale bars are 50 µm. Statistical significance was determined by an unpaired two-tailed Student’s *t*-test (* = *p* < 0.05, ** = *p* < 0.01, *** = *p* < 0.001).

In order to further elucidate the binding interactions between cells and JCol, a binding inhibition assay was performed. The HT-1080s, HFFs and MSCs were incubated with function-blocking antibodies against β1 integrin subunit or against αVβ3 integrin to block binding to any exposed RGD sites. Heparin sodium salt was also used to block binding to the cell surface heparan sulfate chains (Figure 9).

As previously shown, the HT-1080 cells did not bind to JCol, even in the absence of binding inhibition. The application of a β1 subunit blocking antibody to the HT-1080 cells cultured on RTC produced a drastic reduction in adhesion, consistent with the role of the α2β1 integrin in HT-1080 binding to collagen. The HFF cells showed a similar adhesion profile to the HT-1080 cells on RTC; however, a significant reduction in adhesion to JCol was observed when heparin was included (approximately 75% reduction in cell number). This suggests that heparan sulfate chain binding plays a much greater role in JCol-mediated adhesion than for mammalian collagens. This effect was also recapitulated by the MSCs, although to a lesser degree (approximately 50% reduction). Representative images of the cells that managed to attach showed a greatly reduced cell area for the HFFs and MSCs on JCol when heparin sodium salt was included. This reduction in cell area was not observed on the RTC coatings.

The effect of the other individual inhibitory treatments was also less noticeable for MSCs compared with the other cell types (although still significant), possibly due to a more versatile adhesosome. MSC adhesion was only completely ablated when β1 integrin subunit blocking was combined with αVβ3 integrin blocking or with heparin binding inhibition. The blocking of αVβ3 integrin produced a small reduction in adhesion for HFFs and MSCs, suggesting that RGD sequences are present in both RTC and JCol, but are not major motifs for adhesion.

## 3. Discussion

The abundance and diversity of marine invertebrate collagens make them interesting as alternative biomaterial sources for research and industry [24]. Not only can the ethical and religious constraints of mammalian products be circumvented through alternative sources, but there are undoubtedly future applications that could be developed based on their unique biological properties.

As demonstrated here and elsewhere, there are major differences between jellyfish collagens and mammalian collagens, such as their amino acid composition, thermal stability and cell adhesion interactions [31,49,50,51,52].

### 3.1. SDS-PAGE Analysis of JCol

SDS-PAGE analysis revealed several major protein bands for JCol, which is in agreement with previous publications on the molecular weight of the collagens extracted from *R.pulmo* [33,49]. However, it was also observed that JCol contained several faint bands, which may be due to the presence of degradation products formed during extraction or to remnant non-collagenous proteins [53]. A slight increase in molecular weight was also observed in the densest bands when compared with the α chains of RTC. This is most likely due to differences in the chain length and amino acid composition between JCol and RTC, as reported elsewhere [50,51]. SDS-PAGE analysis is only a cursory examination into the collagens present within a sample. Proteomic analysis will eventually be necessary to determine the true collagen composition of JCol and to establish the identity of the different bands (see the discussion on jellyfish collagenomes below). As shown by Tian et al., the cuticle of the sea cucumber *Apostichopus japonicus* contains two fibrillar collagens, as well as two fibril-associated collagens with interrupted triple helices (FACIT), which were not detected through SDS-PAGE but, rather, through proteomic analysis [54].

### 3.2. JCol Secondary Structure and Thermal Stability

The UV CD spectrum of JCol is consistent with the triple-helical structure typical of collagen. The melting temperature of JCol was determined to be approximately 29.1 °C, which is similar to a previously reported value of 28.9 °C [49]. A melting temperature of 35.3 °C was also previously reported [52], although this value was determined visually by observing a reduction in the CD spectra peaks between separate scans at 4.0–4.7 °C intervals, which is a less accurate technique than measuring ellipticity at a fixed wavelength whilst continuously increasing the temperature. We also observed a slight degree of refolding immediately after thermal denaturation. It is possible that a greater degree of trimeric re-assembly would occur if given more time, or if using a more physiologically relevant buffer than ultrapure water, although the extent of the refolding efficiency is unknown.

### 3.3. JCol Fibrillogenesis

Our findings on the fibrillogenesis of JCol are similar to those of Hoyer et al., who used collagen derived from *Rhopilema esculentum* [55]. We found that increasing the ionic strength by using NaCl or NaH_2_PO_4_ increased the solubility of JCol after neutralization. This was verified through AFM imaging, which showed less fibrillar precipitation at high ionic strengths. However, it was also observed for both salts that particular concentrations (i.e., 100 mM NaCl and 25 mM NaH_2_PO_4_) enabled the optimal formation of fibers despite the lower proportion of insoluble collagen available, as measured using the fibrillogenesis assay. It may be that fibrillogenesis requires a balance between ionic strength and free soluble collagen to optimally facilitate the molecular interactions involved in fiber formation. It is also possible that jellyfish tissues contain a different ionic equilibrium to mammals, given the high salinity of their natural habitat, which, through evolution, may have led to different physiological requirements for optimal fibrillogenesis.

The prolonged incubation time only improved fibrillogenesis under suboptimal conditions (high or low ionic strength), and generally did not benefit the fibers that had already formed after 4 h. Varying the temperature had a minimal effect on the proportion of collagen precipitation at either time-point. Incubation temperatures of 4 °C and 20 °C were deemed appropriate given the low melting temperature of this collagen.

### 3.4. Mammalian Cell Adhesion to JCol

The inability of the HT-1080 cells to effectively adhere to JCol conflicts with one previous study, in which viable cells were reported to adhere to JCol for 8 days [49]. A possible explanation for this variation may be that there are differences in the purification methodologies utilized by any research group and a commercial provider of tissue-extracted collagens, such as JCol shown in this study. HT-1080 cells rely almost exclusively on α2β1 integrin for cell adhesion [47,48]. Thus, our inferred finding is that JCol does not contain, or possesses very few, α2β1 integrin binding sites as HT-1080 adhesion was reduced approximately 90% on JCol compared to RTC. Further investigation using a binding inhibition assay revealed that HFF adhesion to JCol was greatly inhibited (approximately 95% reduction) by blocking the function of β1 with an anti-β1 integrin subunit antibody. This would suggest that other collagen-binding β1 integrins may play a major role in fibroblast adhesion to JCol, namely α1β1, α10β1 and α11β1 [56,57,58]. This finding is curious given the overlap in collagen motifs identified for both α1β1 and α2β1 integrins (e.g., GFOGER, GMOGER, GLOGER, etc.) [59]. It may be that JCol contains a higher proportion of motifs unique to α1β1 binding or that the less common α10β1 and α11β1 integrins perform the principal integrin interaction with JCol. In principle, JCol may contain partially unfolded zones with exposed RGD sites that could interact with the RGD-binding β1 integrins α5β1, αVβ1 or α8β1 [60]. However, the small reduction in HFF and MSC adhesion to JCol after αVβ3 integrin blocking suggests that any exposed RGD sequences in JCol are not the principal contributors to cell adhesion.

The addition of the anti-β1 subunit blocking antibody significantly reduced cell adhesion to both RTC and JCol. The effect on MSC adhesion was less pronounced than on HFF or HT-1080 cell adhesion, although it was still significant. This suggests that MSCs are less dependent on the adhesion mechanisms based on β1 integrins and possess a more versatile adhesosome.

A significant adhesion interaction between JCol and the heparan sulfate chains was observed. JCol was retained in immobilized heparin columns, and the addition of soluble heparin resulted in a significant reduction in cell adhesion to JCol, presumably through binding to JCol and competing with the interaction between JCol and the membrane-bound heparan sulfate chains. Heparan sulfate chains perform a role in cell adhesion by binding to proteins through physicochemical means, such as electrostatic attraction to lysine and arginine residues, as well as through non-covalent interactions [61]. Given that the addition of heparin had no effect on RTC but caused a significant reduction in adhesion to JCol by both HFFs and MSCs (approximately 75% and 50% reduction, respectively), it is likely that JCol contains sequences that bind to heparan sulfate or proteoglycans in general.

The differences in the cell adhesion interactions presented by JCol versus mammalian collagens could have a major effect on integrin-mediated signal transduction, gene expression and cell phenotype when using JCol as a substrate for cell culture and differentiation. For example, chondrocyte phenotype maintenance is highly dependent on integrin-mediated SMAD signaling for chondrogenic gene expression and the use of JCol as a substrate for cartilage regeneration may incur different responses to mammalian collagens [62,63]. The effect of a reduced cell area for MSCs cultured on JCol compared to RTC may be an indication of reduced mechanotransduction, which is also a major factor in signaling pathway initiation and, ultimately, in determining the cell phenotype.

### 3.5. Jellyfish Collagenomes

Marine invertebrates do not share the same levels of collagen sequence conservation found across mammals and vertebrates in general. Thus, it does not seem appropriate to classify them following the conventional collagen ‘type’ nomenclature [5,6]. This system of collagen classification was developed from the study of mammalian collagens and is highly dependent upon collagen amino acid sequences determined at the genetic level. Furthermore, mammalian collagen types are defined so that heterotrimers typically occur between genetically distinct chains from the same type, such as α1(I) and α2(I) in type I collagen. Mammalian collagen genes do not correspond directly to those found in known marine invertebrates’ genomes (or currently unknown in the case of *R. pulmo*). Thus, it is inaccurate to definitively claim that specific marine invertebrate collagen genes or proteins belong to types ‘X’ or ‘Y’ as is frequently written in the literature. We agree with the suggestion by Zhang et al. that it may be more appropriate to switch to Arabic numerals, as opposed to Roman numerals, in the nomenclature of invertebrate collagen genes, such as the Hcol1 name used for the first discovered fibrillar collagen in *Hydra vulgaris* [64].

As the genome of *R. pulmo* has yet to be sequenced, it is difficult to precisely determine the exact number and identity of the collagen chains that are being co-purified during the isolation of JCol, or the molecular reasons for their biophysical attributes. However, it is possible to examine the genomes of other medusozoa (such as scyphozoa and hydrozoa) in order to roughly gauge the extent of collagen gene conservation between vertebrates and jellyfish species overall.

A number of biochemical, genomic and transcriptomic analyses of jellyfishes and hydrozoans have been reported over the last decade. These studies aim to address questions on animal evolution, the origins of the jellyfish body plan, or the patterns of gene expression at different life cycle stages of the animals [39,65,66,67,68]. The most extensive research into the genetic origins of medusozoan collagens has been conducted using *H. vulgaris*, a representative hydrozoan organism. Early research by Deutzmann et al. demonstrated the presence of proteins with collagen sequences in the ECM of Hydra mesoglea, namely Hcol1, a fibrillar homotrimer of 155 kD chains, and a 290 kD Type IV collagen-resembling protein that was appropriately named Hcol4 [69]. Later work by Zhang et al. demonstrated the presence of a further three fibrillar collagen genes in *H. vulgaris*, named Hcol2, Hcol3 and Hcol5, and another network-forming collagen named HCol6, with different domain architectures [64].

**Figure 10:**
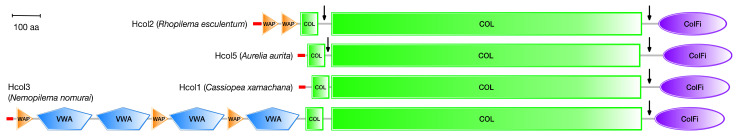
Domain architectures of representative collagen-like sequences from different jellyfish genomes as predicted by the SMART protein domain annotation server (https://smart.embl-heidelberg.de (accessed on 21 November 2022) [70]. All fibrillar collagens contain a C-terminal fibrillar collagen domain (ColFi) of 220-230 amino acids, a major collagen (COL) domain of typically 1023 amino acids, and a minor COL domain of typically 57 amino acids. Hcol2 sequences contain 1 or 2 four-disulfide core domains (WAP), whereas Hcol3 sequences contain three WAP domains intercalated with four von Willebrand factor type A domains. The vertical arrows indicate regions of potential proteolytic cleavage during post-translational processing, based on the similarity with mammalian procollagen sequences. The red tips indicate predicted signal peptide sequences. Domains are drawn to scale to their lengths (scale bar = 100 amino acids).

Other medusozoan genomes are at a relatively early stage of annotation, but it is possible to take an initial inventory of the sequences containing the characteristic repetitive sequences of triple-helical collagen domains (a more detailed study on medusozoan collagenomes will be presented elsewhere). The initial number of collagen-like sequences in these genomes varies between approximately 25 to 70, depending on the different methods used to automatically annotate the genomic data. Many of these sequences are incomplete, or are fragments of longer collagen genes, but it is often possible to reconstruct their complete sequence by analyzing the genome data in more detail. Based on the sequence similarity and domain architecture, several collagen sequences in the jellyfish genomes can be related to the fibrillar or network-forming collagens from *H. vulgaris* (Table 1 and Figure 10).

Hydra and other medusozoan fibrillar collagen sequences share several characteristics with mammalian fibrillar procollagens (Figure 10): a major collagen triple-helical domain (COL) of typically 1023 amino acids with few or no interruptions; a minor COL domain of typically 57 amino acids N-terminal to it; and a C-terminal trimerization domain homologous to those seen in vertebrate fibrillar procollagen sequences (ColFi). Additional domains appear in Hcol2 and Hcol3 sequences (VWA, WAP, Figure 10) and in other fibrillar sequences not discussed here. Network-forming collagen sequences (Hcol4 and Hcol6) are characterized by long COL domains with multiple interruptions and a C-terminal tandem repeated domain that is homologous to that seen in type IV mammalian collagens. Hcol6 sequences also contain additional WAP and VWA domains (not shown).

Nevertheless, medusozoan collagen sequences show low levels of conservation to mammalian sequences. Sequence analysis of the major COL domain of Hcol1 shows a less than 50% similarity to the respective domains in murine α1(I), α2(I) or α1(II) collagen chains [64]. Given that every third amino acid in the Gly-X-Y tripeptide repeats of the major triple-helical domains is glycine, the actual similarity in the X and Y amino acid positions is approximately 15%. A similar level of sequence disparity to vertebrate collagen is observed for other invertebrate collagens and is expected for the collagens of all jellyfishes, in general, and of *R. pulmo* in particular. This poor similarity further supports the argument for not categorizing marine invertebrate collagens as types I, II, III, etc., as is conducted for mammalian and vertebrate collagens in general, which display much higher levels of sequence conservation in their procollagen genes. Interestingly, a preliminary analysis of the amino acid sequences of the jellyfish collagens show that they do not contain the GFPGER motifs (GFOGER after prolyl hydroxylation) recognized by α2β1 integrins, which would be consistent with the lack of adhesion of JCol to the HT-1080 cells reported here (Figure 8).

The Hcol1 collagen from *H. vulgaris* is reported to be a homotrimer [64]. On the other hand, biochemical analyses of the fibrillar collagens extracted from the mesoglea of several jellyfishes suggest heterotrimer formation, with either three distinct chains, α1, α2 and α3 (*N. nomurai*, *S. meleagris*) [71,72], or two chains, α1 and α2, in an approximate 2:1 ratio (*R. esculentum, R. pulmo, A. aurita*; see Figure 1) [30,49]. As shown in Table 1, scyphozoan genomes contain at least seven fibrillar collagen genes, of which Hcol1, Hcol2 and Hcol5 have very similar domain architecture, particularly if post-translational processing occurs (Figure 10). It is likely that some of these genes will correspond to the different α collagen chains visible in the SDS-PAGE analyses of jellyfish mesoglean collagens, including *R. pulmo* (Figure 1).

Mammalian fibrillar collagen genes code for procollagen molecules that need to be processed post-translationally to become mature collagen chains capable of forming the characteristic collagen fibers. During this processing, the C-terminal ColFi domains (C-propeptides) are removed by the action of procollagen C-proteinases, and the chains are further cleaved by procollagen N-proteinases between the minor and major COL domains [5,6]. The domain architecture of some of the jellyfish collagen genes suggests that a similar process may take place (Figure 10). It is known, however, that the N-terminal propeptide of Hydra Hcol1 is retained after post-translational processing. This propeptide probably interferes with fibrillogenesis and causes the reduction in the fiber diameter seen for this collagen [64]. A similar effect may also be responsible for the reduced fiber diameter shown here and elsewhere for *R. pulmo* [73]. The linker region between the minor and major COL domains in Hcol1 collagens is very short, which could be the reason for the retention of their N-terminal propeptides.

## 4. Materials and Methods

### 4.1. Collagen

Collagen derived from *R. pulmo* was purchased from Jellagen (Cat: JL10ML-4, Cardiff, UK). The RTC was purchased from Merck KGaA (Cat: C3867, Darmstadt, Germany). Both the JCol and RTC were solubilized in 20 mM acetic acid to stock concentrations of 3.9 mg/mL and 3 mg/mL, respectively, and stored at 4 °C.

### 4.2. SDS-PAGE

Samples for sodium dodecyl sulfate-polyacrylamide gel electrophoresis (SDS-PAGE) were prepared by adding 15 µL of NuPAGE LDS sample buffer (Cat: NP0007, Thermo Fisher Scientific, Waltham, MA, USA) to 5 µL of the acid-solubilized collagen stock solutions. The samples were then denatured by heating to 95 °C for 5 min before 15 µL aliquots were pipetted to each well of a 4–12% NuPAGE Bis-Tris mini protein gel (Cat: NP0321, Thermo Fisher Scientific, Waltham, MA, USA). A 4 µL aliquot of pre-stained molecular weight standard (Cat: 1610374, Bio-Rad Laboratories, Hercules, CA, USA) was included in each gel. Gel electrophoresis was conducted at 15 mA using a Mini-Protean II Cell (Bio-Rad Laboratories, Hercules, CA, USA) for 90 min.

Non-specific protein staining was performed by staining the gels overnight at 4 °C with a Coomassie protein staining solution (Cat: ISB1L, Abcam, Cambridge, UK). Excess stain was removed by washing and soaking in water for 10 min before the gels were imaged.

Periodic acid-Schiff (PAS) staining was performed by fixing the gels in 50% MeOH for 30 min, soaking in 0.5% periodic acid (Cat: 3803812, Leica Microsystems, Wetzlar, Germany) for 5 min, staining in Schiff reagent (Cat: 3803800E, Leica Microsystems, Wetzlar, Germany) for 10 min and finally clearing with 3% acetic acid for 10 min before imaging. The gels were washed three times in ultrapure water between every step.

### 4.3. Circular Dichroism Spectroscopy

The CD spectra were obtained using a Jasco J-810 Spectropolarimeter (JASCO UK Ltd., Heckmondwike, UK). The JCol samples were diluted to 0.5 mg/mL in ultrapure water and kept on ice prior to measuring. Aliquots of 500 µL were pipetted into quartz cuvettes (0.1 cm path length, Starna Scientific, Ilford, UK) and molar ellipticity measurements were taken between 190 nm and 260 nm. Ultrapure water alone was used as a buffer baseline and subtracted from all of the JCol spectra.

The melting curve of the JCol was plotted by measuring the molar ellipticity at 220 nm at every 0.2 °C increment during a temperature gradient of 0.5 °C/min from 10 °C to 60 °C. The spectra were measured at 10 °C, 60 °C and again at 10 °C immediately post heat denaturation to show triple helix refolding. The melting temperature (Tm) of the JCol was calculated using the non-linear regression analysis function in Graphpad Prism software. A 4 parameter symmetric sigmoidal curve was fitted to the plot of the change in ellipticity as a function of temperature (T) [74].

### 4.4. Heparin Chromatography

Heparin-affinity chromatography was performed using a 1 mL HiTrap Heparin HP column (Cat: 17040601, Cytiva, Marlborough, MA, USA) on an AKTAprime FPLC chromatography system (Cytiva, Marlborough, MA, USA). First, the column was equilibrated with 15 volumes of running buffer (10 mM NaH_2_PO_4_, pH 7.4). The samples were prepared by buffering and neutralizing 650 µL JCol with 250 µL 100 mM Tris buffer (pH 7.4) and 100 µL 200 mM NaOH. The neutral buffered collagen was filtered using a 40 µm syringe filter (Cat: FC122, Sartorius AG, Göttingen, Germany) and 500 µL of filtered sample was then injected into the chromatography system at a flow rate of 0.5 mL/min. The elution commenced 20 min post-injection using running buffer with 2 M NaCl over a 15 min linear gradient (0–100%). A fraction size of 1 mL was used when collecting flow-through for SDS-PAGE analysis as described previously (Section 4.2).

### 4.5. Collagen Fibrillogenesis Assay

One milliliter aliquots of neutral-buffered JCol was created by buffering 650 µL aliquots of acid-solubilized JCol stock with 250 µL of 100 mM tris-(hydroxymethyl)-aminomethane (Tris) buffer and neutralizing with 100 µL of 200 mM NaOH. The samples were kept on ice between handling and a pH of 7.2–7.4 was verified using pH indicator strips (Cat: HC900874, Merck KGaA, Darmstadt, Germany). The NaCl buffer solutions were diluted to 1.67× required molarity in 25 mM Tris before adding 150 µL of each to 100 µL aliquots of neutral-buffered JCol. This produced 250 µL aliquots of 1 mg/mL JCol samples supplemented with the required NaCl concentration (0–500 mM). The samples were incubated at 4 °C or 20 °C for 4 h and 24 h before pipetting 15 µL from each sample into a 96-well plate in duplicate. The remaining 220 µL from each sample was centrifuged at 15,000 G for 15 min at 4 °C. After centrifugation, 15 µL was collected from the supernatant solution of each sample into a 96-well plate in duplicate.

To determine the proportion of fibrillogenesis, a bicinchoninic acid assay (Cat: 23235, Thermo Fisher Scientific, Waltham, MA, USA) was performed by adding 235 µL of the working solution to each sample, incubating at 37 °C for 1 h and measuring the absorbance at 575 nm using a microplate photometer (Multiscan FC, Thermo Fisher Scientific, Waltham, MA, USA). Protein concentrations from the supernatant samples were subtracted from corresponding values obtained prior to centrifugation to infer the proportion of non-solubilized (fibrillar) collagen. Dilutions of the collagen stock were used as a BCA standard instead of albumin.

### 4.6. Atomic Force Microscopy

The collagen samples fibrillated at 20 °C were pipetted (100 µL) onto glass coverslips and left for 30 min before rinsing 5 times with ultrapure water and left to dry at room temperature overnight before imaging. AFM images were acquired on a Multimode 8 AFM (Bruker, Billerica, MA, USA) with a Nanoscope IV controller (Bruker, Billerica, MA, USA) and Nanoscope v8.15 software (Bruker, Billerica, MA, USA). Imaging was performed in ScanAsyst mode in air at a scan rate of 1Hz and a sample rate of 512 on 10 µm^2^ regions.

### 4.7. Cell Culture

The human foreskin fibroblast (HFF) cells were purchased from ATCC (Cat: SCRC-1041, ATCC, Manassas, VA, USA) and cultured in Dulbecco’s Modified Essential Medium Eagle (DMEM) (Cat: D0819, Merck KGaA, Darmstadt, Germany) containing 10% fetal calf serum (FCS) (FCS-SA, Labtech International Ltd., Heathfield, UK), 100 U/mL penicillin with 100 µg/mL streptomycin (P4333, Merck KGaA, Darmstadt, Germany), 1% GlutaMAX supplement (Cat: 35050038, Invitrogen, Waltham, MA, USA) and 1 mM sodium pyruvate (Cat: S8636, Merck KGaA, Darmstadt, Germany).

The human fibrosarcoma (HT-1080) cells were purchased from ATCC (Cat: HT-1080, ATCC, Manassas, VA, USA) and cultured in DMEM containing 10% FCS, 100 U/mL penicillin with 100 µg/mL streptomycin and 1% GlutaMAX supplement.

The immortalized human bone marrow-derived mesenchymal stem cells (Y201) were gifted to the research group by Professor Paul Genever from the University of York (UK). These cells originate from a human MSC population infected via lentivirus to overexpress hTERT enzyme and maintain key MSC hall marks such as multi-lineage differentiation potential and mechanosensitivity [75,76]. These cells were cultured in Minimum Essential Medium Eagle (MEM) (Cat: M4526, Merck KGaA, Darmstadt, Germany) containing 10% FCS, 100 U/mL penicillin with 100 µg/mL streptomycin, 1% GlutaMAX supplement and 10 µM Asc-2-Phos (A2P) (Cat: A8960, Merck KGaA, Darmstadt, Germany), and were cultured between a passage number of 75 and 85.

### 4.8. Proliferation and Spreading Assay

The acid-solubilized collagen stocks were diluted to 20 µg/mL in phosphate buffered saline (PBS) (Cat: D8537, Merck KGaA, Darmstadt, Germany) and coated onto 12 well plates overnight at 4°C. The well plates were rinsed with PBS before blocking with 1% bovine serum albumin (BSA) (Cat: 11021-029, Thermo Fisher Scientific, Waltham, MA, USA) in PBS for 60 min.

For the proliferation assay, the cells were seeded at a density of 10,000/cm^2^ in replicate plates (N = 3) and collected every 24 h for immunofluorescence staining and quantitative imaging (Section 4.9). For the spreading assay, the cells were suspended in medium without serum before seeding at a density of 25,000/cm^2^ and replicate plates were collected every 24 h.

### 4.9. Immunofluorescence Staining and Quantitative Imaging

For the experiments that utilized cell imaging for generating quantitative data, the cells were fixed at appropriate time-points using 4% paraformaldehyde (PFA) for 30 min before washing three times in PBS. Wells were then blocked using 1% BSA for 45 min, washed with PBS and stained with 1/400 Alexa Fluor™ 488 phalloidin (Cat: A12379, Thermo Fisher Scientific, Waltham, MA, USA) and 4 µM (1/5000) Hoescht 33342 solution (Cat: 62249, Thermo Fisher Scientific, Waltham, MA, USA) for 45 min. The wells were then washed three times with PBS before immediately imaging under a fluorescence microscope (Olympus BX51, Olympus UK and Ireland, UK) under a 4X objective lens.

For each collagen coating, cell type and time-point 9 images were taken at different regions across 3 replicate wells in a 12-well plate. Using the image analysis software FIJI [77], the cell number present in each image was quantified by converting the 8-bit image using a binary threshold and automatically counting the number of particles within range of 300–5000 µm^2^ and circularity of 0.65–1. The average cell area could then be calculated by dividing the cumulative cell area present in a thresholded image by the number of particles counted.

### 4.10. Adhesion Assay

Collagen-coated 12-well plates were prepared and blocked as described previously (Section 4.8). The cells were seeded at 25,000/cm^2^ in media without serum to minimize non-specific adhesion. After 24 h, the plates were collected for immunofluorescence staining and quantitative imaging (Section 4.9).

### 4.11. Binding Inhibition Assay

The acid-solubilized collagen stocks were diluted to 20 µg/mL in PBS and coated onto 96 well plates overnight at 4 °C. The well plates were rinsed with PBS and blocked with 1% BSA/PBS for 60 min at room temperature before rinsing with PBS. Prior to cell seeding, cells were suspended at 1 × 10^6^/mL in media without serum before 350 µL aliquots were pipetted into Eppendorf tubes containing either 7 µL of anti-β1 integrin subunit antibody (Cat: MABT821, Merck KGaA, Darmstadt, Germany) at 500 µg/mL (10 µg/mL final), 1.75 µL of anti-αVβ3 integrin antibody (Cat: MAB1976, Merck KGaA, Darmstadt, Germany) at 1 mg/mL (5 µg/mL final), 7 µL of heparin sodium salt (Cat: H3149, Merck KGaA, Darmstadt, Germany) at 50 mg/mL in ultrapure water (1 mg/mL final) or combinations of all 3 treatments.

All of the binding inhibition reactions were incubated at 37 °C for 30 min before adding 50 µL/well (16,000/cm^2^) in 3 replicate wells for each collagen coating. Serial dilutions of the untreated cell stock were also seeded onto both collagen coatings in order to create a standard of known cell number for later quantification of relative adhesion for treated groups.

The cells were allowed to adhere for 2 h before washing with PBS and fixing in 4% PFA for 30 min. Wells were then washed three times in PBS and stained with 0.1% aqueous Crystal violet solution (Cat: V5265, Merck KGaA, Darmstadt, Germany) for 1 h. After washing away excess dye three times with PBS, cells were lysed with 100 µL 10% acetic acid for 15 min on an orbital shaker. The absorbance of the released dye was then measured at 575 nm using a microplate photometer.

### 4.12. Statistical Calculations

All of the graphs display a mean value ± standard deviation. Statistical significance was determined by an unpaired two-tailed Student’s *t*-test (* = *p* < 0.05, ** = *p* < 0.01, *** = *p* < 0.001).

### 4.13. Preliminary Analysis of Collagen Sequences in Jellyfish Genomes

Collagen-like sequences from several jellyfish genomes [39,64,65,66,68,69,78,79,80,81] were identified using in house scripts, detecting the repetitive Gly-X-Y sequences characteristic of the collagen triple helical domains. Most of the initial hits were incomplete sequences or corresponded to fragments of longer collagen genes. When possible, more complete sequences were reconstructed through detailed analysis of the DNA genome data. The jellyfish collagens were classified on the basis of their sequence similarity to Hydra collagens and their domain architectures as predicted by SMART [70]. A more comprehensive analysis of the jellyfish collagenomes will be presented elsewhere.

## 5. Conclusions

This article provides a background characterization of the collagen derived from the jellyfish *R. pulmo*. We demonstrate some of the key similarities and differences between the collagen derived from this marine invertebrate and a mammalian fibrillar collagen commonly used in biomedical applications.

Based on the biophysical characterization and human cell adhesion data on JCol shown here, as well as the variation in the collagen gene sequences found in other medusozoa, it is reasonable to suggest that these collagens are not directly interchangeable alternatives to the mammalian collagens. Marine invertebrate collagens present their own unique biomaterial properties and, in the case of JCol, can be used for further research as substrates for atypical collagen-cell adhesion and, potentially, phenotype regulation. Only once these collagens have been sequenced and fully characterized can they properly inform the design of future products and cell scaffolds for biomedical research.

## Figures and Tables

**Table 1:** Numbers of fibrillar and network-forming collagen-like sequences in the genomes of different medusozoa, classified by the corresponding orthologues in the *Hydra vulgaris* genome.

Genome	Class	HCol1, HCol2, HCol5	Hcol3	Other fibrillar	Hcol4	Hcol6
*Hydra vulgaris*	Hydrozoa	3	1	2	1	1
*Clytia hemisphaerica*	Hydrozoa	5	1	2	1	4
*Aurelia aurita*	Scyphozoa	4	1	2	1	1
*Nepomilema nomurai*	Scyphozoa	4	1	2	1	1
*Rhopilema esculentum*	Scyphozoa	4	1	3	2	1
*Chrysaora quinquecirrha*	Scyphozoa	4	1	2	1	1
*Cassiopea xamachana*	Scyphozoa	4	1	2	1	1
*Cassiopea andromeda*	Scyphozoa	4	1	2	1	1
*Alatina alata*	Cubozoa	2	1	2	1	1
*Morbakka virulenta*	Cubozoa	2	1	2	1	2
*Calvadoxia cruxmellitensis*	Staurozoa	2	1	2	1	1

## Data Availability

Data generated during this research is included in the published article. Any reasonable requests for additional information can be provided through correspondence with the authors.
